# More relevant than ever: Celebrating a decade of *Preventive Medicine Reports*^☆^

**DOI:** 10.1016/j.pmedr.2025.103339

**Published:** 2025-12-05

**Authors:** Eduardo L. Franco

**Affiliations:** Division of Cancer Epidemiology, McGill University, Montreal, QC, Canada

When we had the privilege of launching *Preventive Medicine Reports* (PMR), as founding co-editors, we titled our inaugural editorial in 2014 (Franco and Rosberger, 2014) “Is there a need for a new journal devoted to preventive medicine?” It was an obvious question to ask, given the fluidity of scholarly publishing in the health sciences, some 15 years after the birth of the open-access model with successful pioneers such as BioMed Central and the Public Library of Science (PLOS). The consequent surge in research productivity and the transformative impact of open-access publishing in democratizing access to scientific knowledge, particularly for resource-constrained countries, was unequivocal. We introduced PMR as a new open-access journal dedicated to advancing disease prevention, health promotion, and public health policy. PMR adopted a sound-science peer-review model, inspired by PLOS One, launched in 2006, to publish ethically conducted, methodologically robust studies regardless of perceived novelty. PMR aimed at addressing gaps in specialized and regional preventive medicine research and fostering knowledge translation. As an offshoot of *Preventive Medicine*, PMR inherited the credibility of its parent journal but maintained the same rigorous peer review, leveraging Elsevier's infrastructure and an experienced editorial team.

Although we were imbued with a sense of an editorial mission to serve the public health research community in 2014, we wanted to be sure that PMR was a good fit with the modern framework for the professional practice of preventive medicine. We invited Olli S. Miettinen, an epidemiologist who earned his rightful place in any anthology of the discipline as a brilliant and often controversial thought leader, to write a guest commentary on the meaning of preventive medicine. The late Miettinen (1936–2022) did not disappoint (Miettinen, 2014). He redefined preventive medicine as the prevention of all forms of ill-health—not only disease but also defects, injuries, and sickness unrelated to somatic anomalies. He argued that epidemiology is not merely fundamental but constitutes the community-level segment of preventive medicine. He distinguished preventive medicine from public health and health promotion, advocating for a libertarian approach centered on informed choice rather than authoritarian regulation. He posited that core principles include providing expert guidance to clients and focusing on freedom from sickness rather than prolonging life. The article challenged conventional views, urging conceptual clarity and ethically grounded practices in preventive medicine.

PMR emerged in the above context of self-awareness, questioning its value to the research and practice communities. Was it successful as a journal? Given its rapid growth, with the number of new submissions approaching that of its 50-year-old parent journal, the answer is a resounding yes: over 3000 papers have been published since launch. It has earned a well-deserved reputation in the field, ranking highly across most metrics used in scholarly publishing (Franco, 2022). After 10 years of unequivocal success, it was fitting to celebrate the occasion with a special issue that reunites eight papers representative of PMR's breadth and scope. These articles are available via open access in Elsevier's Science Direct platform.

The eight papers span diverse contexts and a wide range of preventive medicine themes, reflecting the multidimensional nature of preventive medicine in public health and clinical practice. The papers address infectious disease prevention, behavioral health, chronic disease management, and psychosocial well-being, illustrating how preventive strategies extend beyond traditional vaccination to encompass lifestyle, equity, and mental health.

Two studies focused on immunization and vaccine acceptance and uptake among vulnerable populations. Both examined the challenges of administering a proven public health intervention in people living with HIV, including herpes zoster vaccination (Motet et al., 2025) and maternal immunization during pregnancy (Touré et al., 2025). These works highlight barriers such as limited awareness, safety concerns, and cultural influences, underscoring the need for proactive communication by healthcare providers and tailored interventions to improve coverage.

The theme of behavioral risk factors and health disparities was also represented in this celebratory collection. A study of tobacco use and cravings among sexual and gender minority adolescents revealed persistent disparities driven by social stressors and targeted marketing (Azagba et al., 2025). Similarly, a systematic review of physical activity barriers and facilitators among youth highlighted structural and motivational challenges, calling for standardized tools and global strategies to promote active lifestyles (Sampaio et al., 2025).

This collection also underscored the notion that technology can assist health promotion in important ways. Innovative interventions using wearable devices for physical activity among middle schoolers demonstrate feasibility but limited short-term effectiveness, suggesting that family engagement and age-appropriate digital tools are critical for sustained behavior change (Kwon et al., 2025).

Not surprisingly, given the era we just survived, the special issue also included a study of the large population-level stressors on health behaviors. The COVID-19 pandemic's disruption of schooling provided insights into how virtual learning influences children's movement behaviors, screen time, and sleep patterns. The study's findings advocate for integrated approaches that balance educational needs with the promotion of physical and mental health (Prajapati et al., 2025).

Two other papers focused on psychosocial adjustment and chronic conditions. A qualitative study on stroke survivors in Singapore reveals the emotional complexity of coping and adjustment, shaped by cultural expectations and life-stage challenges. Recommendations include embedding mental health support and resilience-building into rehabilitation pathways (Cruz Gonzalez et al., 2025). An investigation of abdominal aortic calcification (AAC) and cardiovascular risk factors revealed that higher AAC scores were strongly associated with age, hypertension, diabetes, and dyslipidemia, indicating its potential as a marker for systemic atherosclerosis. Early AAC detection through imaging could help identify individuals at elevated cardiovascular risk, enabling timely lifestyle interventions and risk factor management to prevent adverse outcomes (Rehemuding et al., 2025).

Collectively, these eight studies are emblematic of the fact that prevention is not limited to disease avoidance but encompasses health equity, behavioral change, psychosocial support, and health services resilience. Effective preventive strategies require multilevel interventions—combining clinical guidance, community engagement, technology, and culturally sensitive approaches—to address both physical and mental health across the lifespan. Could we have asked for a more representative sample of studies for this special issue? I think not; they are a veritable microcosmos of what PMR intends to be, as enshrined in our Aims & Scope: “*… a scholarly repository for the building blocks of research that inform research, practice and policy on disease prevention and health promotion …*”.

The US government has drastically reduced support for research on diversity, equity, and inclusion in health care access. It has also raised unreasonable challenges to the proven value of immunizations in preventing illness. Unfazed, PMR continues to hold the fort with its original mission to provide policymakers with credible scientific evidence to eliminate disparities and improve health. In these uncertain times for public health, PMR is thus more relevant than ever.

## Disclosure

The author served as Editor-in-Chief of *Preventive Medicine* from January 2013 to December 2022 and of *Preventive Medicine Reports* from June 2014 to December 2022. He is grateful to Dr. Luisa Borrell, the current Editor-in-Chief of both journals, for inviting him to curate the present special issue and to write this editorial.
